# Affective observation guides expectations about others’ emotional reactions to unfamiliar action outcomes

**DOI:** 10.1007/s00426-025-02206-0

**Published:** 2025-11-11

**Authors:** Thomas Ganzetti, Luke McEllin, Fabrice Clément, Günther Knoblich

**Affiliations:** 1 Social Mind and Body group (SOMBY), Social Mind Center, Department of Cognitive Science, Central European University, Vienna, Austria; 2 Cognitive Science Center, University of Neuchâtel, Neuchâtel, Switzerland

## Abstract

Observing others’ emotional expressions facilitates individuals’ expectations about the evaluations shared within a social group. This is useful for interpreting ambiguous cultural content, such as unfamiliar actions performed by others. This study investigated whether observed emotional reactions to instrumental actions are used to predict novel individuals’ evaluations of those same actions (i.e., generalization), and whether they are more likely to be generalized for unfamiliar compared to familiar actions. Participants were presented with emotional expressions of observers reacting to individuals performing instrumental actions, before selecting the reaction they expected from a novel observer watching the same action performed by a new individual - generalization meaning that the novel observer was expected to elicit the same emotional expression as the initial observer. Experiment 1 found that negative reactions are generalized to predict negative evaluations of unfamiliar but not familiar actions, whereas Experiments 2 and 3 demonstrated differences in generalization and influence on expectations of positive and negative displays depending on whether familiar and unfamiliar actions were present. This study sheds light on the affective components of cultural learning, showing that the observation of others’ emotional displays enables us to interpret unfamiliar social situations in the absence of direct communication.

**Supplementary Information:**

The online version contains supplementary material available at 10.1007/s00426-025-02206-0.

## Introduction

### Observational learning of actions and punishment avoidance

Observational learning involves learning associations between others’ actions and their outcomes, allowing individuals to efficiently learn an appropriate response to a stimulus based on the observed outcomes of an observed action (reward or punishment) (Kang et al., 2021). This type of learning enables individuals to extract relevant information from the environment while avoiding the costs and risks involved in direct interaction with others (Cavanagh et al., 2014; Olsson et al., 2018). Through observational learning, individuals can safely acquire cultural information necessary for coordinating with other group members, to signal their value within a community, and to behave according to the groups norms and conventions, thereby mitigating risks associated with inappropriate behavior (Ensminger et al., 2014). Observational learning relies on the understanding of an action kind and its association with a certain response in the environment (Kang et al., 2021), which eventually guides individuals to either faithfully replicate the action when it is rewarded or to avoid it when it is punished (Lindström & Olsson, 2015).

Importantly, learning to avoid harm through the observation of punishments may be even more crucial than learning to imitate behaviours that lead to rewards. This is because avoiding negative reactions from others is essential for survival (Selbing & Olsson, 2017) and maintaining group membership (Seymour et al., 2007). Individuals indeed often exhibit heightened sensitivity to potential negative outcomes, which leads them to a prioritization of punishment avoidance over seeking rewards (Lindström & Olsson, 2015). Furthermore, compared to observed positive feedback (Jones et al., 2011), negative feedback tends to have a stronger impact on social behaviour. This makes observational learning a critical mechanism for internalizing the negative consequences of actions without directly experiencing them.

### Importance of emotions and affective displays in cultural learning

Observational learning is particularly relevant when considering the significance of emotional displays, which are signals (Keltner et al., 2019; Shariff & Tracy, 2011) that help regulate our social environment and influence others’ cognition and behaviour (e.g., angry reactions can elicit guilt and lead to prosocial behaviour) (Baumeister et al., 2007; Campos et al., 2006; Parkinson, 1996).

Specifically, emotional expressions are crucial tools for navigating complex cultural environments (e.g., EASI theory) (Van Kleef, 2009; Van Kleef & Côté, 2022), as they can disambiguate social situations, facilitate interpersonal coordination, and support learning (Van Kleef et al., 2011). Depending on the context and the observer’s prior-knowledge, the information retrieved by observing these emotional displays can be used to form evaluations about (a) the target of the emotional reaction, (b) the expresser of the emotional display, or (c) the norms and practices shared by a social group (Hareli & Hess, 2012; Hess & Fischer, 2014).

The above discussion suggests that emotional displays are informative about other individuals’ expectations and evaluations, allowing one to anticipate others’ reactions to an event, especially when faced with uncertain, ambiguous, and unfamiliar situations (Fischer, 2019; Hess et al., 2019). This is particularly important considering that our social environment is laden with behaviours such as rituals, conventional social practices and specific ways of behaving, which can appear “cognitively opaque” (Csibra & Gergely, 2011) because such kind of cultural knowledge is incomprehensible for a naïve observational learner. Importantly, generalization - the capacity to understand specific instances (like the evaluation of an object or action) as representative of broader, universally accepted cultural evaluations, recognized and shared by all the members of a social group (i.e., universally shared cultural knowledge) (Gergely et al., 2007) – facilitates the learnability and the transmissibility of cultural content (Sperber, 1990, 1996).

Specifically, interpreting acquired information as being generalizable to all members of a social group implies that all the individuals of that group are expected to share the same evaluation of a particular target or to perform an action in a certain manner (Egyed et al., 2013), i.e., generalizing a specific instance to many members of a social group allows learners to predict the culturally shared values and ways of doing present in a community, even for individuals they have never encountered. This process facilitates expectations about others’ evaluations within a social group, enabling individuals to identify cultural practices and to determine which behaviours to imitate and which ones to avoid due to the risk of punishment (Csibra & Gergely, 2011). This ability underpins the effective transmission of cultural values across generations and helps individuals to navigate the social environment while minimizing the risks of punishment and ostracization.

Although the influence on learners’ expectations and the generalizability of social information about the values and ways of doing shared by others has been primarily investigated in relation to direct communication (i.e., information acquired in a teaching context is perceived as being generalizable to all the individuals of a social group) (Csibra & Gergely, 2011), observational learning from emotional displays may offer an alternative route for naïve individuals to access opaque culturally shared information. This is particularly the case given that people actively seek out for and pay more attention to emotional cues expressed by others to assess the emotional significance of an event when it is novel and unfamiliar, compared to situations that are familiar and well-known (Bruder et al., 2014). In fact, in unfamiliar contexts, emotional signals quickly convey socially relevant information and thus serve as powerful learning tools (Manstead & Fischer, 2001).

### Observational learning and emotional displays in unfamiliar contexts

By interpreting others’ affective expressions, observers can evaluate unfamiliar cultural content and figure out what is expected from them. The Affective Social Learning account of cultural transmission (ASL), as outlined by Clément and Dukes (2022), emphasizes that any facial expression conveying emotional valences (e.g., positive or negative) can shape the cultural learning of novice members of a social group. Furthermore, while all emotional expressions provide valuable insights for navigating a cultural environment, the ability to anticipate and avoid punishment is crucial in cultural learning (Lindström & Olsson, 2015). In fact, although positive emotional displays inform about rewarding behaviour, they are less salient and differ significantly in their implications (i.e., lack of threat component) compared to negative emotional displays, which inform observers about others’ negative evaluations of a target, enabling to detect potentially dangerous responses (Shariff & Tracy, 2011). Therefore, the observation of negative emotional displays may be especially important for evaluating unfamiliar cultural content. For instance, the detection of an angry face directed at a specific action performed by another individual can help observers to infer the prevalent social practices shared by the group (Hareli & Hess, 2012), even before any first-hand interaction and in absence of direct communication (Manstead et al., 2019).

This phenomenon, termed “affective observation” (Clément & Dukes, 2022) and “emotional eavesdropping” (Repacholi & Meltzoff, 2007), refers to the process by which an individual actively seeks out relevant information by observing third-party interactions, specifically focusing on emotional cues (Dukes & Clément, 2019). In this process, called “observation of affects”, there is not necessarily an intentional attempt by an individual to convey specific information to the observer; rather, the observer actively seeks to acquire information from the emotional displays of others. Thus, by observing an emotional display elicited by other individuals’ actions, a third-party observer can form judgements about the appropriateness of an action (e.g., an angry reaction might signal the violation of a cultural practice) relying only on simple mechanisms (e.g., associative learning), without the necessity of higher-level mechanisms typically involved in teaching contexts, such as complex inferences based on the retrieved mental states of the emotion expressers (Dukes & Clément, 2019).

Furthermore, if social information retrieved during affective observation is perceived as generalizable to other members of a social group (i.e., shared cultural knowledge), it would enable the observer to form expectations regarding a novel individual’s evaluations, ultimately leading to a modulation of behaviour based on observed emotional cues (i.e., punishment avoidance).

This underscores the need for a systematic investigation of how emotional information retrieved from third-party observation of interactions (i.e., affective observation) can help predict individuals’ evaluations (i.e., influence on expectation), and whether the observed evaluations extend to other group members (i.e., generalization),. Such a study is crucial for determining whether affective observation can assist in identifying community cultural practices and conventions.

## The present study

Considering that emotional displays provide important learning opportunities in unfamiliar cultural contexts, we investigated whether shared cultural knowledge regarding evaluations of instrumental actions can be acquired through affective observation, by inducing generalized expectations about others’ emotional reactions.

Specifically, this study tested whether the familiarity, or lack thereof, with the action eliciting an observed emotional display influences the expectation about the evaluations shared by other individuals of a social group, and whether such influence on expectations is enough to generalize the observed evaluations to other individuals (i.e., that novel individuals are expected to react with the same emotional display to the action). We chose to focus on affective observation of instrumental actions – that is, the observation of a third-party interaction in which an individual (*expresser*) emotionally reacts (e.g., displays a negative face) to another individual performing an action which involves the manipulation of two objects. This is suitable for investigating generalization processes and their interaction with prior knowledge about the appraisal of social content because: (1) instrumental actions embody the collective methods and practices of a group in relation to technical knowledge (Charbonneau et al., 2023); (2) uncertainty with respect to the end-state of such actions, combined with lack of context, render these actions “culturally opaque” to naïve observers (Gergely et al., 2002).

In this context, generalizing an observed emotional evaluation of an action to a novel individual of a social group entails expecting that a novel individual will display an emotional expression congruent with the one displayed by a previous individual observing the same action (e.g., after observing someone reacting negatively to an action, a novel individual would be expected to also react negatively when observing that action). This would mean that an observed evaluation is generalized when observers expect novel individuals to also share the same evaluation. The present study investigated generalization of emotional expressions conveying both negative (e.g., anger) and positive (e.g., happiness) valences. However, since observational learning is particularly useful for avoiding punishment and negative social consequences (e.g., ostracization or social harm) (Lindström & Olsson, 2015), we primarily focused on emotional reactions expressing a negative valence.

Moreover, we investigated the influence of prior knowledge on the processing of emotional information by comparing scenarios where the observed emotional displays were elicited by instrumental actions involving everyday objects used either in a familiar way to achieve a clear end-state (which do not trigger a strong emotional reaction), or in a way that produced an unfamiliar and “opaque” end-state. Thus, we presented participants with a scenario where they could observe an individual emotionally react to an instrumental action before selecting the reaction they expected from a novel individual seeing the same action.

To investigate whether opaque learning contexts increase the tendency of observers to rely on negative emotional cues to predict novel individuals’ evaluations, Experiment 1 tested whether participants, after observing a negative emotional reaction, are more likely to expect new individuals to also react negatively compared to when they did not observe a negative reaction (i.e., influence on expectation), and whether they generalize negative reactions (after observing a negative reaction, novel individuals are expected to also display a negative reaction) more often when the observed reaction was elicited by an unfamiliar action compared to a familiar one (Exp. 1). Our key hypothesis was that, although negative emotional displays may influence expectations regarding novel individuals’ evaluations for both familiar and unfamiliar actions, lack of familiarity with the action facilitates the tendency to generalize the observed negative evaluation to novel individuals, as emotional information becomes particularly relevant in the absence of a pre-existing evaluation of the action. However, when familiarity with the action conflicts with unexpected reactions (i.e., incongruent with an expectation generated by a pre-existing evaluation), the influence on expectation would not be sufficient to generalize negative evaluations of that action to new individuals (i.e., the shift in expectation is not sufficient to be systematically extended to new individuals).

Furthermore, to investigate whether lack of familiarity influences generalization of positive and negative emotional reactions, Experiment 2 tested how people process positive and negative emotional displays in response to familiar and unfamiliar actions and if there are differences in the tendency to generalize based on the type of valence. Here, regarding the interaction between type of valence (positive or negative) and learning context (familiar or unfamiliar), we expected negative emotional displays to influence expectations regarding novel individuals’ evaluations, being generalized for unfamiliar but not familiar actions (like for Experiment 1). Moreover, because negative emotional reactions inform about potential punishment, we predicted they would be generalized in unfamiliar contexts regardless of the presence of familiar actions and positive displays. In contrast, positive emotional displays, which are less salient and may signal mere appreciation for the successful outcome of an action, may be systematically expected for familiar actions but yield ambiguous interpretations for unfamiliar actions. This would lead to a decreased tendency to generalize positive reactions to predict novel individuals’ positive reactions in the unfamiliar context, due to their perceived lack of clear relation to culturally shared evaluations.

Finally, to investigate how positive valences can be used in opaque learning contexts to form anticipations about novel individuals’ evaluations, Experiment 3 tested whether both positive and negative emotional displays elicited by unfamiliar actions influence expectations regarding the evaluations of novel individuals and whether they both lead to generalization of observed evaluations. We hypothesized that, when considering only unfamiliar actions, both positive and negative displays may be informative about the environment, thus influencing expectations regarding novel individuals’ evaluations and leading to generalization. However, we did not exclude potential differences in the use of positive and negative displays when predicting others’ emotional reactions.

## Experiment 1

Experiment 1 tested whether people generalize observed negative emotional expressions elicited by instrumental actions, i.e., after observing a negative reaction to an action, people expect new individuals to also react negatively when the same action is performed by another individual. Moreover, we investigated whether people are more likely to generalize the expectation of a negative reaction in novel individuals when the observed negative display is elicited by an unfamiliar compared to familiar action. The Experiment included a neutral baseline (observed neutral reactions instead of negative ones) to ensure that unfamiliar actions are not expected to automatically elicit negative evaluations. The neutral baseline also allowed us to measure influence of negative displays in familiar and unfamiliar actions (i.e., how often a negative emotional display is expected after observing either a negative or a neutral display),

We predicted observed negative displays to influence expectations about novel individuals’ evaluations both for familiar and unfamiliar actions, meaning that novel individuals are expected to display a negative reaction significantly more often after observing a negative display compared to a neutral one. However, we predicted participants to generalize the observed negative evaluations to novel individuals (i.e., after observing a negative reaction, they expect a congruent negative reaction in novel individuals significantly above chance) only after observing negative reactions to unfamiliar but not familiar actions, with a significant difference between the two types of action (measured as percentage of congruent choices). This is because for unfamiliar actions, the observed emotional reaction would not conflict with prior knowledge about the action, thus making it more likely to be used to form an evaluation of the action that could be extended (i.e., generalization) to other individuals.

Regarding the neutral baseline, we predicted participants to expect a neutral reaction more consistently for familiar actions compared to unfamiliar ones, as the neutral reaction is the most appropriate for a familiar action which usually does not trigger negative responses, thus reflecting our manipulation. For the observation of neutral responses to unfamiliar actions, unlike what hypothesized for negative reactions, we did not predict generalization of neutral reactions to novel individuals (i.e., expectation of congruent neutral reactions above chance), since neutral responses do not convey a specific valence and are thus less informative regarding the evaluation of an unfamiliar action.

## Method

### Participants

The final sample for this experiment included 24 participants (Females = 15, *M*_*age*_ = 27.51 years, *SD* = 5.15). The experiment was conducted in our lab; participants were recruited through the university recruitment system SONA, the criteria to participate included English language proficiency and no diagnosed attentional disorders. All participants provided written informed consent prior to their inclusion in the study, in accordance with the protocol approved by the institutional ethics committee. Participants received monetary compensation (10€) via bank-transfer, in accordance with the university policy. Since the study was exploratory, our sample size was determined to ensure adequate sensitivity to detect large effects. Specifically, we considered both the 2 × 2 Repeated measures ANOVA relevant to our hypothesis regarding an effect of familiarity (which required 16 participants to detect an effect of η² = 0.14 with 80% power at α = 0.05) and the two tailed one-sample *t*-tests used to assess generalization of observed reactions (which required 20 participants to detect an effect of *d* = 0.8 with 80% power at α = 0.05/4, Bonferroni-corrected). Because the *t*-tests required more participants to reach 80% power, we adopted this more conservative benchmark. We collected data from 24 participants to ensure enough power in case of potential exclusions. Post-hoc power analyses showed that our study was sufficiently powered to detect differences in generalization of negative and neutral evaluations between familiar and unfamiliar actions (1-beta = 0.99; *d* = 1.26; fixed alpha = 0.05).

### Materials

**Fig. 1:**
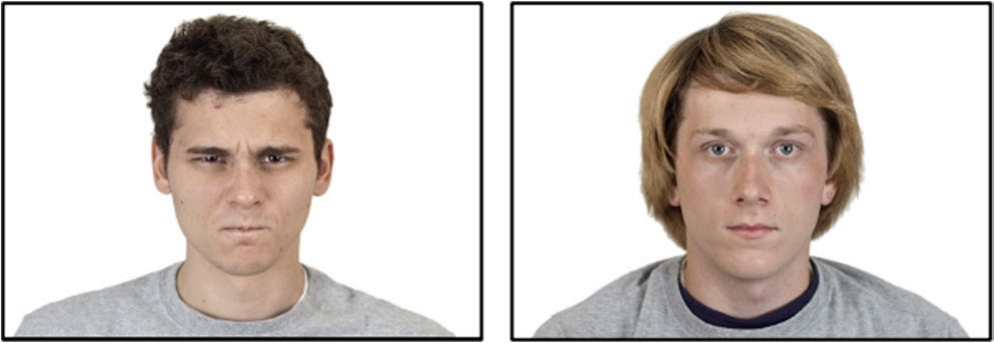
Example of a negative (left) and neutral (right) expresser

Pictures of action performers, negative expressers (i.e., individuals showing angry facial expressions) and neutral expressers (i.e., individuals showing neutral facial expressions) were retrieved from the Chicago face database (https://www.chicagofaces.org) (Ma et al., 2015) (Fig. 1). 240 different individuals were selected from the database (160 action performers, and 80 expressers displaying negative reactions and 80 expressers displaying neutral reactions), for a total of 320 pictures (size: 295 × 211px), evenly split by gender and ethnicity (“black” and “white”, as categorized in the database).

**Fig. 2:**
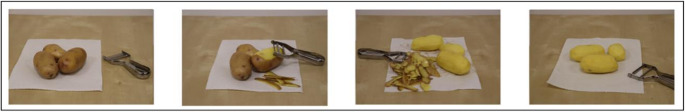
Example of a familiar action. Peeling potatoes

**Fig. 3:**
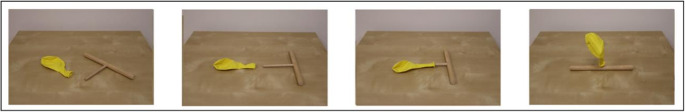
Example of an unfamiliar action. Putting a stick in a balloon

The sequences of pictures resembling the instrumental actions were taken in our lab, each involving the manipulation and interaction of two objects on a table. Each action was captured in a series of four pictures (size: 300 × 211px) representing different stages of the action as it unfolds. Familiar actions consisted in everyday common actions with familiar goals and means (e.g., Fig. 2), whereas unfamiliar actions were characterized by an unclear and unfamiliar end-state (e.g., Fig. 3). None of the unfamiliar actions included elements associated with negative responses (e.g., an end-state producing damage or breaking the object). All the material, including the entire set of actions, can be downloaded from the following link: https://osf.io/uh4y3/?view_only=db53af7df0e042b49385050147388440.

### Procedure

**Fig. 4:**
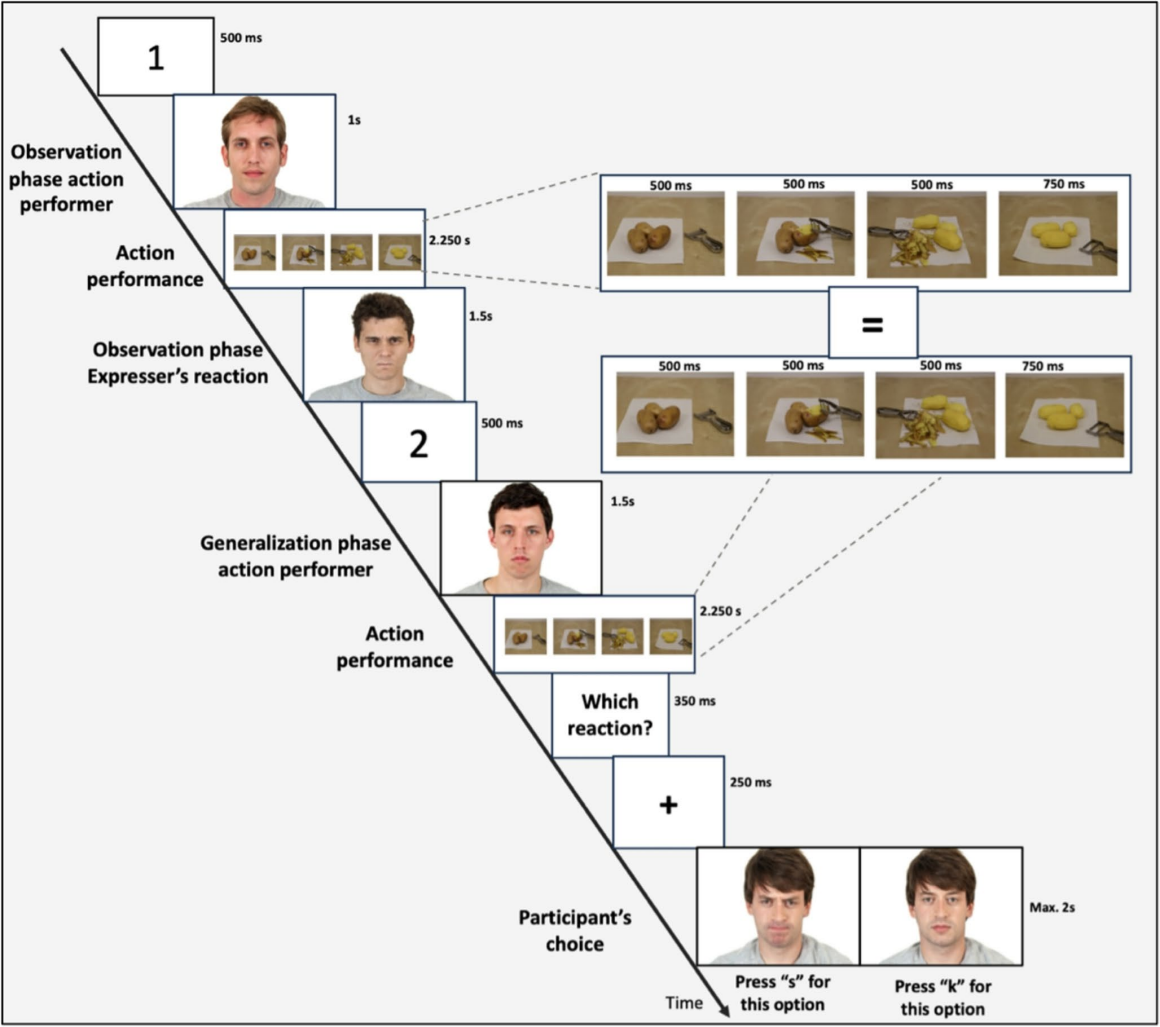
Graphical example of one trial. In the observation phase, the participant is presented with an individual (observation phase performer) performing an instrumental action and another individual (observation phase expresser) reacting to it either with a negative or neutral emotional display. In the generalization phase, a new individual (generalization phase performer) performs the same action, and the participant selects which reaction they expect a new expresser (generalization phase expresser) will display. Video examples can be found in the supplementary material

For our task (see Fig. 4 for a graphical overview), each trial consisted of an observation phase followed by a generalization phase.

The observation phase began with the number “1” appearing at the center of the screen for 500ms, followed by a 500ms pause. Participants were then presented with a picture of the individual who would perform the action (observation phase performer) accompanied by the label “performer of the action” (displayed for 1000ms). This was followed by a 700ms pause and then a sequence of 4 pictures representing each stage of the action involving two objects accompanied by the label “action performance”. These stages were timed as follows: “stage 1” for 500ms, “stage 2” for 500ms, “stage 3” for 500ms, and “end-state of action” for 750ms. The end-states were either clear (familiar) or “opaque” (unfamiliar). After the first action, participants saw the picture of another individual (observation phase expresser) reacting either negatively (negative condition) or neutrally (neutral condition) to the action, accompanied by the label “observer’s reaction” (displayed for 1500ms).

The generalization phase began with the number “2” appearing at the center of the screen for 500ms, followed by a 500ms pause. Participants were then shown a picture of a new individual who would perform the action (generalization phase performer) accompanied by the label “performer of the action” (all timing and stimulus sizes were the same as in the observation phase), followed by the same pictures resembling the action observed in the observation phase (i.e., to show the second performer completing the action). After the action had been shown, the prompt “which reaction?” appeared at the center of the screen for 350ms followed by a 200ms pause, asking participants to prepare to indicate which emotion they expect a new individual (generalization phase expresser) will display. A fixation cross then appeared for 250ms followed by a 100ms pause. Next, two pictures of the generalization phase expresser showing the two potential emotional displays appeared on the two sides of the screen, one showing a negative reaction and one showing a neutral reaction. These were pseudorandomized to appear equally on the left and right sides in a random order. Participants chose the expected display by pressing “s” (for the left) or “k” (for the right) on their keyboard. If the choice took more than 2000ms, the message “too slow” was shown in red capital letters for 500ms. After responding there was a 500ms pause before the beginning of the next trial. It was crucial to use pictures that clearly showed different individuals (observation phase performer; observation phase expresser; generalization phase performer; generalization phase predicted expresser) to ensure that the prediction of the emotional reaction in the generalization phase expresser would be related to an emotional evaluation of the action itself and to an emotional evaluation of the individual performing the action.

All experiments presented in this manuscript were conducted in accordance with the Declaration of Helsinki and were approved by the ethics committee of Central European University, Vienna.

Participants completed 80 trials divided in two blocks separated by a 30-second break. In each block, 20 trials depicted a familiar action (familiar condition), and 20 trials depicted an unfamiliar action (unfamiliar condition). In each condition, the action elicited a negative reaction in one half of the trials, and a neutral reaction in the other one. Before starting the Experiment, participants completed two practice trials at a slower speed.

At the end of the Experiment, participants rated the familiarity of the actions on a 7 point Likert scale (“How familiar is the outcome of this action?”; 1 = “Not familiar at all”, 7 = “Very familiar”), and the intensity of the negative emotional expressions on a 7 point Likert scale (“How would you rate the intensity of this emotional expression?”; 1 = “Not intense at all”, 7 = “Very intense”). Finally, participants were presented with the Emotional Contagion Scale (ESC) (Doherty, 1997). Please see supplementary material for data on participants’ subjective categorizations of action familiarity, perceived intensity of emotional displays, and sensitivity to emotional cues.

The average duration time of the experiment was approximately 40 min.

The order of the actions, the combinations of expressers and performers, and the position of negative and neutral reactions (left and right choice on the screen) were randomized. Furthermore, every trial included exclusively individuals of the same gender and ethnicity to avoid that actors and reactors were perceived as being part of different cultural groups. Additionally, the order of the ratings related to subjective actions’ familiarity and emotional expressions’ intensity was counterbalanced across participants. The set of 80 individuals displaying an emotional reaction was repeated in the two blocks: their pairing with the different actions and whether they appeared in the observation phase or in the generalization phase was randomized for each participant. Each action from our action set appeared only once per participant, without repetitions.

### Design and data analysis

This Experiment employed a 2 × 2 within-subjects design, with type of action (familiar or unfamiliar), and the valence of the emotional reaction (neutral or negative) as factors.

We had two dependent variables: the first was the expectation of a negative reaction in the generalization phase observer, independently of the emotional display present in the observation phase. The second dependent variable was the congruency between the generalization phase expresser’s expected reaction and the observation phase expresser’s reaction (i.e., whether the generalization phase expresser was expected to react like the observation phase expresser). For each participant, we calculated the percentage of expected negative reactions and percentage of expected congruent reactions in each condition (40 trials for the familiar condition, half neutral and half negative; 40 trials for the unfamiliar condition, half neutral and half negative).

First, to determine whether participants’ expectation of a negative reaction was influenced by the reaction displayed in the observation phase (neutral or negative), we conducted a 2 × 2 repeated measures ANOVA with valence (negative, neutral) and type of action (familiar, unfamiliar) as within-subjects factors, and percentage of expected negative reactions as the dependent variable. Furthermore, to determine whether the negative reaction of the first expresser significantly influenced the prediction of the reaction displayed by the second expresser for familiar and unfamiliar actions respectively, we performed paired-sample t-tests comparing the percentage of expected negative reactions after seeing a negative reaction and after seeing a neutral reaction (neutral baseline).

Second, to determine whether participants generalized the observed reactions to novel expressers, we performed a 2 × 2 repeated measures ANOVA with valence (negative, neutral) and type of action (familiar, unfamiliar) as within-subjects factors, and percentage of congruent expectation as the dependent variable.

Moreover, we conducted two one-sample t-tests to check whether the percentage of congruent expectation in both the familiar and unfamiliar condition was above chance level (i.e., 50%), meaning that the observed emotional reaction was used to systematically expect the same reaction in new individuals (i.e., generalization). Additionally, to test whether there were any differences between the familiar and unfamiliar actions, we performed a paired sample t-test comparing percentage of congruent expectation when observing familiar actions versus unfamiliar actions.

When appropriate, we also performed Bayesian tests to evaluate the evidence for the null hypothesis relative to the alternative hypothesis. For all the Bayesian tests performed, we used a Cauchy prior with a scale of 0.707 (this is a conventional default in Bayesian testing frameworks; Rouder et al., 2009).

Furthermore, we used participants’ ratings of perceived familiarity with the actions to create two subjective categories: actions perceived as being familiar (rating ≥ 4), and actions perceived as being unfamiliar (rating < 4). Please see supplementary material for analyses with subjective categorizations of the actions. All data and analyses are available on the following link: https://osf.io/uh4y3/?view_only=db53af7df0e042b49385050147388440.

We excluded 3 (out of 1920) trials with reaction times less than 200ms or greater than 3s.

## Results

### Manipulation check and neutral baseline

We performed a chi-squared test to ensure that participants’ subjective categorization of the actions accurately reflected our manipulation. The test revealed that there was a significant relationship between participants’ categorizations and our manipulation (*X*^*2*^*(1)* = 967.87, *N* = 1840, *p* < 0.001, *V* = 0.71). Regarding our set of unfamiliar actions, participants rated them as being unfamiliar 77.2% of the time. For familiar actions, participants rated them as familiar 93% of the time. Additionally, participants’ ratings of perceived intensity of the reactions encountered show a significant difference between negative displays (*M = 4.73, SD = 1.14) and neutral displays (M = 1.75, SD = 0.58) (t(23) = 14.15, p < 0.001, d = 0.93*), confirming the correct interpretation of the facial displays.

In line with our manipulation, in the absence of a negative reaction (neutral baseline) familiar actions were expected to trigger a negative reaction significantly below chance level (*M* = 13.43%, *SD* = 12.33%) (*t(23) =* −14.53, *p <* 0.001, *d = -*2.97, 95% CI [8.23, 18.64]), indicating a clear expectation based on prior-knowledge (i.e., familiar actions are not expected to trigger negative evaluations). For unfamiliar actions, the neutral baseline showed that the expectation of a negative reaction after seeing a neutral reaction was not different from chance (*M* = 42.71%, *SD* = 25.54%) (*t(23) =* −1,40, *p =* 0.36, *d = -*0.28, 95% CI [31.93, 53.49]). The Bayesian test provided anecdotal evidence that it was at chance level (BF_01_ = 1.969), in line with the idea that unfamiliar actions were characterized by lack of prior-knowledge regarding their evaluation.

### Expectation of negative reactions compared to baseline (influence on expectation)

When looking at the interaction between valence and type of action with respect to expectation of a negative reaction, our 2 × 2 ANOVA revealed a significant main effect of valence (*F(*1,23) = 21.78, *p* < 0.001, *η*_*p*_^*2*^ = 0.49), of type of action (*F*(1,23) = 35.06, *p* < 0.001, *η*_*p*_^*2*^ = 0.60) and no significant interaction between type of action and valence (*F*(1,23) = 2.24, *p* = 0.148, *η*_*p*_^*2*^ = 0.09) on expectation of a negative reaction. When examining the expectation of a negative reaction for unfamiliar actions, we found that they were expected significantly more after observing a negative reaction (*M* = 76.64%, *SD* = 21.29%) compared to a neutral reaction (*M* = 42.71%, *SD* = 25.54%) (*t(23) =* 4.91, *p <* 0.001, *d =* 1.00, 95% CI [19.63, 48.25]). When looking at familiar actions, we also found that negative reactions were expected significantly more after observing a negative reaction (*M* = 42.29%, *SD* = 33.03%) compared to a neutral one (*M* = 13.43%, *SD* = 12.33%) (*t(23) =* 4.14, *p <* 0.001, *d =* 0.86, 95% CI [14.44, 43.28]). We found convergent results when considering participants’ subjective categorizations of actions (see supplementary material).

### Generalization of negative reactions

When examining the interaction between valence and type of action with respect to generalization, our 2 × 2 ANOVA revealed a significant main effect of valence (*F*(1,23) = 7.46, *p* < 0.05, *η*_*p*_^*2*^ = 0.24), no effect of type of action (*F*(1,23) = 2.24, *p* = 0.148, *η*_*p*_^*2*^ = 0.09) and a significant interaction between type of action and valence (*F*(1,23) = 35.06, *p* < 0.001, *η*_*p*_^*2*^ = 0.60) on expectation of congruent reactions.

**Fig. 5:**
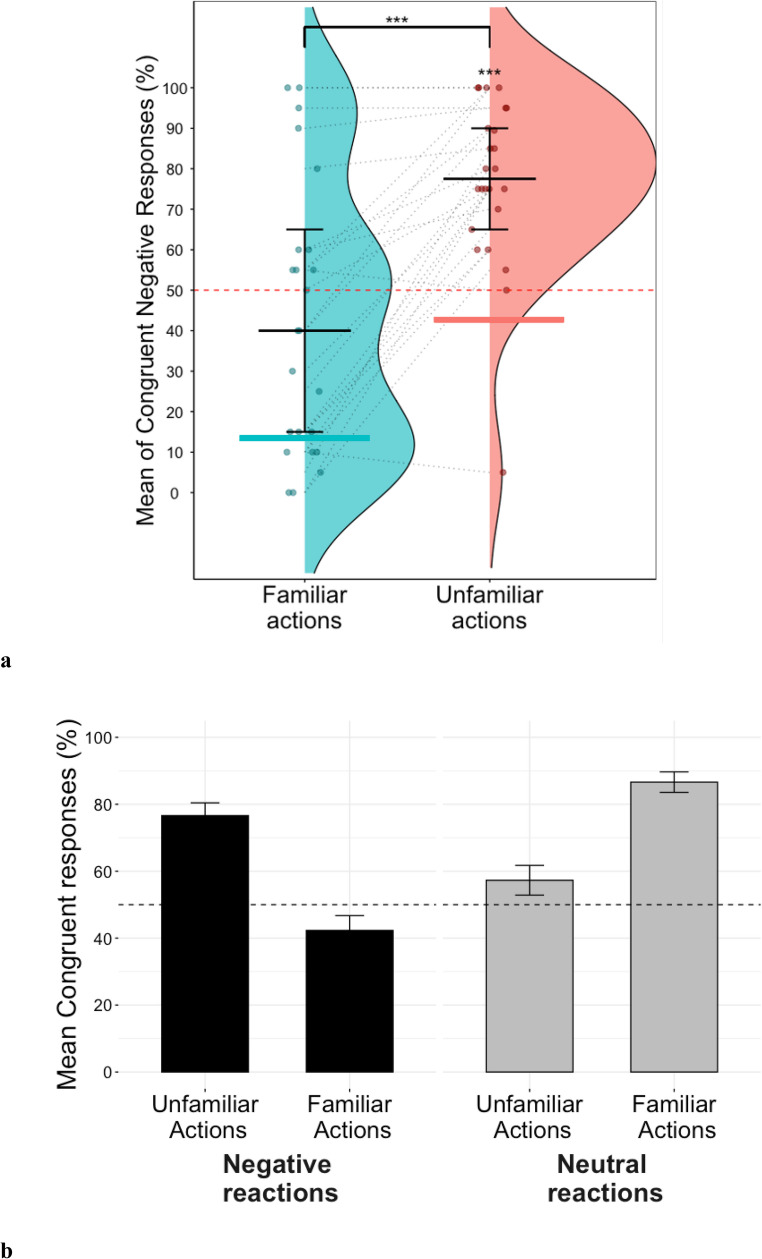
** a** Raincloud plots showing percentage of congruent negative responses for familiar and unfamiliar conditions. Dashed red line indicates chance level (50%). The two coloured segments indicate the baseline for expected negative reactions to familiar (13.43%) and unfamiliar actions (42.71%) respectively. **b** Barplots showing mean percentage of congruent responses for all conditions. Dashed red line indicates chance level (50%)

When examining the differences in expectation of congruent negative emotional displays between familiar and unfamiliar actions, results showed that expectation of congruent negative reactions for unfamiliar actions (*M* = 76.64%, *SD* = 21.29%) was significantly above chance (*t(23) =* 6.133, *p <* 0.001, *d =* 1.25, 95% CI [67.66, 85.63]), which was not the case for familiar actions (*M* = 42.29%, *SD* = 33.03%), (*t(23) = -*1.143, *p =* 0.27, *d =* 0.23, 95% CI [28.34, 56.24]) (Fig. 5). The results of the Bayesian analysis provided anecdotal evidence that participants’ expectation of congruent negative responses for familiar actions was mostly at chance (BF_01_ = 2.602). Moreover, participants tended to generalize more often in the unfamiliar compared to familiar condition (*t(23) =* 6.163, *p <* 0.001, *d = 1.26*, 95% CI [22.82, 45.88]; Fig. 5).

Regarding the differences in congruent expectation of neutral responses to familiar and unfamiliar actions, congruent expectation of neutral responses for unfamiliar actions (*M* = 57.29%, *SD* = 25.54%) was not significantly different from chance (*t(23) =* 1.40, *p* = 0.18, *d =* 0.29, 95% CI [46.51, 68.07]), but was above chance for familiar actions (*M* = 86.57%, *SD* = 12.33%), (*t(23) =* 14.53, *p <* 0.001, *d =* 2.97, 95% CI [81.36, 91.77]). The results of the Bayesian analysis provided anecdotal evidence that participants’ expectation of neutral displays for unfamiliar actions was mostly at chance (BF_01_ = 1.969). Moreover, participants tended to expect congruent neutral reactions in the familiar condition significantly more than in the unfamiliar one (*t(23) =* 5.14, *p* < 0.001,* d = 1.05*, 95% CI [17.50, 41.06]) (Fig. 5). Furthermore, results indicate a significant difference in congruent expectation between negative and neutral reactions for unfamiliar actions (*t(23) =* 2.91, *p <* 0.01, *d =* 0.60, 95% CI [5.60, 33.11]) and for familiar actions (*t(23) =* −5.97, *p <* 0.001, *d* = 1.22, 95% CI [28.94, 59.62]). When considering the subjective categorizations of actions based on participants’ ratings, we found results that converged with our experimental manipulation (see supplementary material for all the analysis).

### Discussion experiment 1

In line with our key hypothesis, negative emotional reactions significantly influenced participants’ expectations of negative reactions in novel observers both for unfamiliar and familiar actions, compared to when they did not observe a negative reaction (neutral baseline). Moreover, participants generalized the negative evaluations to novel individuals only for unfamiliar actions, and not for familiar ones. Lack of familiarity with the action eliciting the reaction led people to rely enough on observed emotional displays to generalize it and expect novel individuals to display congruent negative evaluations (i.e., above chance); on the contrary, when participants were familiar with the action eliciting the reaction, the negative emotional display conflicted with prior evaluations of the action (i.e., usually not triggering negative evaluations), making the influence of the observed negative reactions insufficient to generalize such expectation to novel individuals (i.e., above chance). These results indicate a strong effect of lack of familiarity on the tendency to use observed emotional displays to generalize negative evaluations of an action to novel individuals.

Regarding our neutral baseline, it showed that in the unfamiliar condition, the expectation of neutral reactions in novel observers was not different from chance, thus being insufficient to generalize neutral reactions - people relied on emotional information only when it carried a negative valence, highlighting the saliency and relevance of emotional cues (compared to neutral expressions) in learning about the cultural environment. Moreover, the presence of a neutral baseline ensured that the influence on participants’ expectation and on generalization of negative responses in the unfamiliar condition was not due to lack of familiarity automatically triggering expectations of negative evaluations.

## Experiment 2

Experiment 1 focused on the effect of observed negative emotional displays upon expectations regarding novel individuals’ negative emotional evaluations, and their generalization. However, it is unclear whether these findings also extend to positive emotional displays, which are also important to generalize as they highlight potential rewards. Thus, Experiment 2 investigated whether both negative and positive emotional displays influence predictions about novel individuals’ evaluations of unfamiliar and familiar actions, and whether they are also generalized to novel individuals. Specifically, we aimed at replicating Experiment 1 in relation to negative emotional displays, showing that, although they influence expectations both for familiar and unfamiliar actions, they are generalized only for unfamiliar actions, even in the presence of positive displays. Furthermore, we wanted to investigate whether negative emotional reactions are more likely to be generalized to novel individuals compared to positive ones.

It is particularly important to investigate positive displays in the context of familiar actions, which may render the emotional displays ambiguous. Positive displays may signal mere appreciation for the successful end-state of a familiar instrumental action, thus being less salient for cultural learning and having a less marked conflict with prior knowledge compared to negative emotional displays. The procedure was the same as in Experiment 1, except we presented positive and negative reactions.

We predicted that participants’ expectations would be influenced by negative displays for both familiar and unfamiliar actions, being generalized (i.e., above chance) for unfamiliar actions only. We predicted positive displays to also influence participants’ expectations both for familiar and unfamiliar actions, leading to generalized expectations for positive responses to familiar actions (i.e., holding expectations regarding positive reactions to succesful outcomes of known instrumental actions). Furthermore, since the interpretation for positive displays may be more ambiguous, we considered that participants may be more likely to generalize negative compared to positive reactions regarding unfamiliar actions. Thus, we predicted that participants would expect congruent reactions above chance for positive but not negative reactions to familiar actions, with a significant difference between the two.

## Method

### Participants

The final sample for this experiment included 20 participants (Females = 12, *M*_*age*_ = 24.56 years, *SD* = 3.60). Participant recruitment and sample size rationale were the same as in Experiment 1; since no one met our exclusion criteria in Experiment 1, we proceeded to recruit 20 people as according to our power analysis.

Post-hoc power analyses showed that our study was sufficiently powered to detect differences in generalization between familiar and unfamiliar actions both for negative (1-beta = 0.99; d= 1,01; fixed alpha: 0.05) and positive (1-beta = 0.99; d = 1.27; fixed alpha: 0.05) displays.

### Materials & procedure

**Fig. 6:**
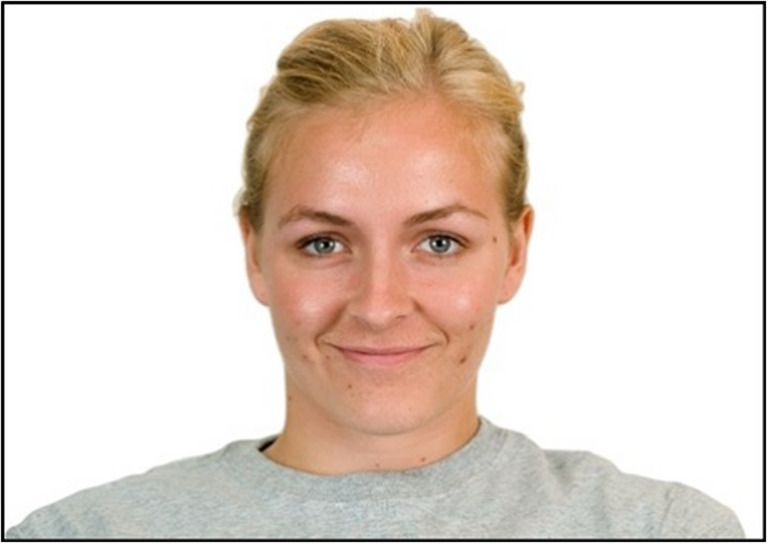
Example of a positive expresser

Materials and procedure were identical to Experiment 1, except the following:

1. The observation phase expresser displayed either a negative or a positive expression (i.e., a happy face – see Fig. 6). All other aspects of the first expresser stimuli (e.g., randomization, counterbalancing) was the same as Experiment 1b.
2. The generalization phase expresser potential displays were either positive or negative (80 trials: 20 negative and 20 positive for familiar and unfamiliar actions).

### Design, coding, and data analysis

This was the same as in Experiment 1, except with positive rather than neutral trials. For influence of positive displays on participants’ expectations, the test results were identical to those for negative displays because the data was reverse-coded. Specifically, since the means of expected negative responses and positive responses were complementary (e.g., expecting a negative reaction 30% of the time meant expecting a positive one 70% of the time), the comparison between positive and negative expectations yielded the same differences regardless of the specific measure used. So, we only reported the means for the expectation of positive reactions in each condition. We excluded 27 (out of 1600) trials with a reaction time less than 200ms or greater than 3000ms.

## Results

### Manipulation check

We performed a chi-squared test to ensure that participants’ subjective categorization of the actions accurately reflected our manipulation. The test showed that there was a significant relationship between participants’ categorizations and our manipulation (*X*^*2*^*(1)* = 501.53, *N* = 1840, *p* < 0.001, *V* = 0.56).

Regarding our set of unfamiliar actions, participants rated them as being unfamiliar 70.38% of the time, while 29.62% of the time they judged them to be familiar. For familiar actions, participants rated them as familiar 85.12% of the time, and unfamiliar 14.88% of the time.

### Expectation of a negative reaction based on the emotion observed (influence on expectation)

When looking at the interaction between valence and type of action with respect to expectation of a negative reaction, our 2 × 2 ANOVA revealed a significant main effect of valence (*F*(1,19) = 21.98, *p* < 0.001, *η*_*p*_^*2*^ = 0.13), of type of action (*F*(1,19) = 18.64, *p* < 0.001, *η*_*p*_^*2*^ = 0.11) and no significant interaction between type of action and valence (*F*(1,19) = 0.03, *p* = 0.866, *η*_*p*_^*2*^ < 0.01) on expectation of a negative reaction. When examining the expectation of a negative reaction for unfamiliar actions, we found that they were expected significantly more after observing a negative reaction (*M* = 78.99%, *SD* = 20.34%) compared to a positive reaction (*M* = 43.67%, *SD* = 27.90%) (*t(19) =* 4.09, *p <* 0.001, *d =* 0.91, 95% CI [17.25, 53.39]). When looking at familiar actions, we also found that negative reactions were expected significantly more after observing a negative reaction (*M* = 42.29%, *SD* = 33.03%) compared to a positive one (*M* = 13.43%, *SD* = 12.33%) (*t(19) =* 3.69, *p <* 0.01, *d =* 0.82, 95% CI [15.54, 55.71]). We found convergent results when considering participants’ subjective categorizations of actions (see supplementary material).

Descriptively, this means that when considering the expectation of a positive reaction for unfamiliar actions, we found that they were expected more often after observing a positive reaction (*M* = 56.34%, *SD* = 27.90%) compared to a negative reaction (*M* = 21.02%, *SD* = 20.34%). This was also the case for familiar actions, with positive reactions being expected more often after observing a positive reaction (*M* = 89.60%, *SD* = 11.71%) compared to a negative one (*M* = 54.06%, *SD* = 38.38%).

#### Generalization of negative and positive reactions

When examining the generalization of observed negative and positive reactions to novel individuals, Our 2 × 2 ANOVA revealed no effects of valence (*F*(1,19) = 0.414, *p* = 0.52, *η*_*p*_^*2*^ = 0.005) or type of action (*F(*1,19) = 0.014, *p* = 0.89, *η*_*p*_^*2*^ = 0.004) on expectation of percentage of congruent expectations, but a significant interaction between type of action and valence (*F*(1,19) = 9.844, *p* < 0.01, *η*_*p*_^*2*^ = 0.12).

**Fig. 7:**
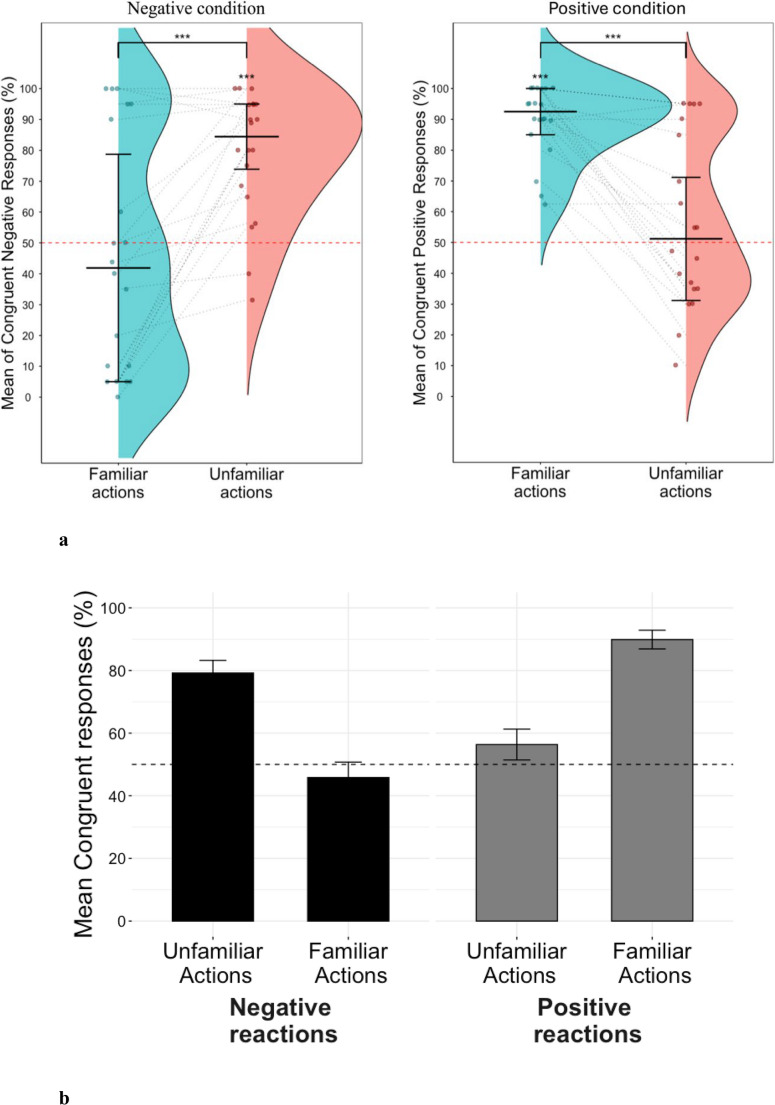
**a **Raincloud plots showing percentage of congruent responses for familiar and unfamiliar conditions. The red line indicates the chance level (50%). **b **Bar plots showing mean percentage of congruent responses for all conditions. Dashed red line indicates chance level (50%)

When examining the differences in generalization of negative emotional displays between familiar and unfamiliar actions, results showed that participants’ congruent expectations for unfamiliar actions (*M* = 78.98%, *SD* = 20.34%) was significantly above chance (*t(19) =* 6.37, *p <* 0.001, *d =* 1.42, 95% CI [69.46, 88.50]), which was not the case for familiar actions (*M* = 45.94%, *SD* = 38.38%), (*t(19) = -*0.47, *p =* 0.64, *d =* 0.11, 95% CI [27.98, 63.90]) (Fig. 7).

The results of the Bayesian analysis provided anecdotal evidence that participants’ expectation of congruent negative responses for familiar actions was mostly at chance level (BF_01_ = 3.891). Moreover, participants tended to expect congruent negative reactions in the unfamiliar condition significantly more than in the familiar one (*t(19) =* 4.53, *p <* 0.001, *d* = 1.01, 95% CI [17.79, 48.30]; Fig. 7).

Regarding differences for expectation of congruent positive responses to familiar and unfamiliar actions, results indicated that congruent expectation for unfamiliar actions (*M* = 56.33%, *SD* = 27.89%) was not significantly different from chance (*t(19) =* 1.016, *p* = 0.32, *d =* 0.23, 95% CI [43.28, 69.39]), but was significantly above chance for familiar actions (*M* = 89.60%, *SD* = 11.71%), (*t(19) =* 15.13, *p <* 0.001, *d =* 3.38, 95% CI [84.13, 95.08]). The results of the Bayesian analysis provided anecdotal evidence that participants’ expectation of congruent positive reactions for unfamiliar actions was mostly at chance (BF_01_ = 2.730). Furthermore, participants expected congruent positive expressions in the familiar condition significantly more than in the unfamiliar condition (*t(19) = 5.67,p < 0.001, d = 1.27*, 95% CI [20.98, 45.55]) (Fig. 7).

Additionally, there was a significant difference in expectation of congruent negative and positive expressions both for unfamiliar actions (*t(19) = 3.39*,* p <* 0.01, *d =* 0.75, 95% CI [8.66, 36.63]) and for familiar actions (*t(19) = −5.29*,* p* < 0.001, *d* = 1.18, 95% CI [26.40, 60.93]). When considering the subjective categorizations of actions based on participants’ ratings, we found convergent results with our experimental manipulation (see supplementary material for all the analysis).

## Discussion experiment 2

Our results extend the findings of Experiment 1 by showing that, even in the presence of positive displays, negative emotional displays influenced expectations about novel individuals’ emotional evaluations both for unfamiliar and familiar actions but led to generalization of negative evaluations to novel individuals only for unfamiliar actions.

In line with our predictions, although positive emotional displays influenced expectations both for familiar and unfamiliar actions (i.e., expecting more positive responses after observing a positive response compared to a negative one), unlike negative reactions they were generalized to new individuals for familiar actions exclusively, indicating that positive displays were likely interpreted as showing appreciation for the successful end-state of familiar actions. Moreover, positive displays were not generalized for unfamiliar actions. This finding aligns with the expected increase in ambiguity concerning the role of positive displays in a context where familiar actions were also present, suggesting that positive displays may also be interpreted as signalling simple feedback regarding the completion of the action and not necessarily convey information about cultural evaluations. In sum, these results indicate that because positive reactions do not strongly conflict with prior knowledge in the way negative reactions do, they are more easily taken as signals of successful action completion, limiting their role in forming expectations about culturally shared evaluations in unfamiliar contexts.

Thus, positive emotional displays may be not always informative for learning about shared cultural evaluations, being more subject to contextual effects, compared to negative displays which seem to consistently and robustly provide valuable information regardless of contextual cues. Finally, it is worth noting that about 30% of participants categorized some unfamiliar actions as familiar. Importantly, however, analyses based on participants’ subjective categorizations (perceived familiar vs. perceived unfamiliar) yielded the same pattern of results as the analyses based on the experimental manipulation (see Supplementary Material), providing further evidence for the robustness of our findings.

## Experiment 3

Experiment 2 showed that positive emotional reactions may be ambiguous in relation to familiar actions, as they could be interpreted as mere appreciation for the successful outcome of a known instrumental action (i.e., they present a less marked conflict with prior knowledge compared to negative emotional displays) rather than as cues to broader cultural evaluations. This suggests that the limited influence of positive displays on participants’ expectations of positive evaluations of unfamiliar actions and their generalization to novel individuals may have been due to the competing interpretation created by familiar actions. To address this ambiguity, in Experiment 3 we focused exclusively on unfamiliar actions. The procedure employed was identical to the previous experiments, except that only unfamiliar actions accompanied by either positive or negative expressions were presented. This design allowed us to test whether positive reactions, when stripped of the familiar-action context, would be generalized in the same way as negative reactions. We predicted that, with such ambiguity removed, participants’ expectations would be influenced by both types of observed expressions - negative and positive - and that they would both be generalized (i.e., above chance) to novel individuals.

Moreover, although we did not have strong predictions about differences in the influence on expectation and generalization regarding positive versus negative reactions to unfamiliar actions, this design provided a way to test for differences without contextual confounds. Specifically, negative expressions, as highlighted in the introduction, are often more salient in cultural learning (i.e., punishment avoidance) and may therefore influence expectations more, potentially being more likely to be generalized to novel individuals compared to positive reactions.

## Method

### Participants

We recruited 20 participants (Females = 11, *M*_*age*_ = 24.44 years, *SD* = 1.73). Recruitment and sample size rationale were the same as in Experiment 2. A post-hoc power analysis showed that we were sufficiently powered to detect an effect of negative (1-beta = 0.99; *d* = 1.24; fixed alpha = 0.05) and positive (1-beta = 0.91; *d* = 0.78; fixed alpha = 0.05) emotional displays on participants’ expectations above chance level (generalization).

### Materials & procedure

Materials and procedure were identical to Experiment 2 except for the following: only unfamiliar actions were displayed, meaning that this Experiment included only 40 trials (20 positive and 20 negative trials) thus took around 30 min.

### Design, coding and data analysis

This was the same as Experiment 2, but with only one within factor: valence (positive or negative).

We calculated our dependent variables in the same manner as in Experiment 2 and performed the same tests conducted for influence on expectation and generalization of evaluations regarding unfamiliar actions.

We excluded 16 (out of 800) trials with a reaction time less than 200ms or greater than 3s.

## Results

### Expectation of a negative reaction based on the emotion observed (influence on expectation)

When looking at the expectation of a negative reaction for unfamiliar actions, we found that they were expected significantly more after observing a negative reaction (*M* = 73.83%, *SD* = 19.49%) compared to a positive reaction (*M* = 29.42%, *SD* = 26.40%) (*t(19) =* 4.96, *p <* 0.001, *d =* 1.11, 95% CI [25.68, 63.14]).

Descriptively, this means that when examining the expectation of a positive reaction for unfamiliar actions, we found that they were expected significantly more after observing a positive reaction (*M* = 70.58%, *SD* = 26.40%) compared to a negative reaction (*M* = 26.17%, *SD* = 19.48%).

### Generalization of positive and negative reactions

**Fig. 8:**
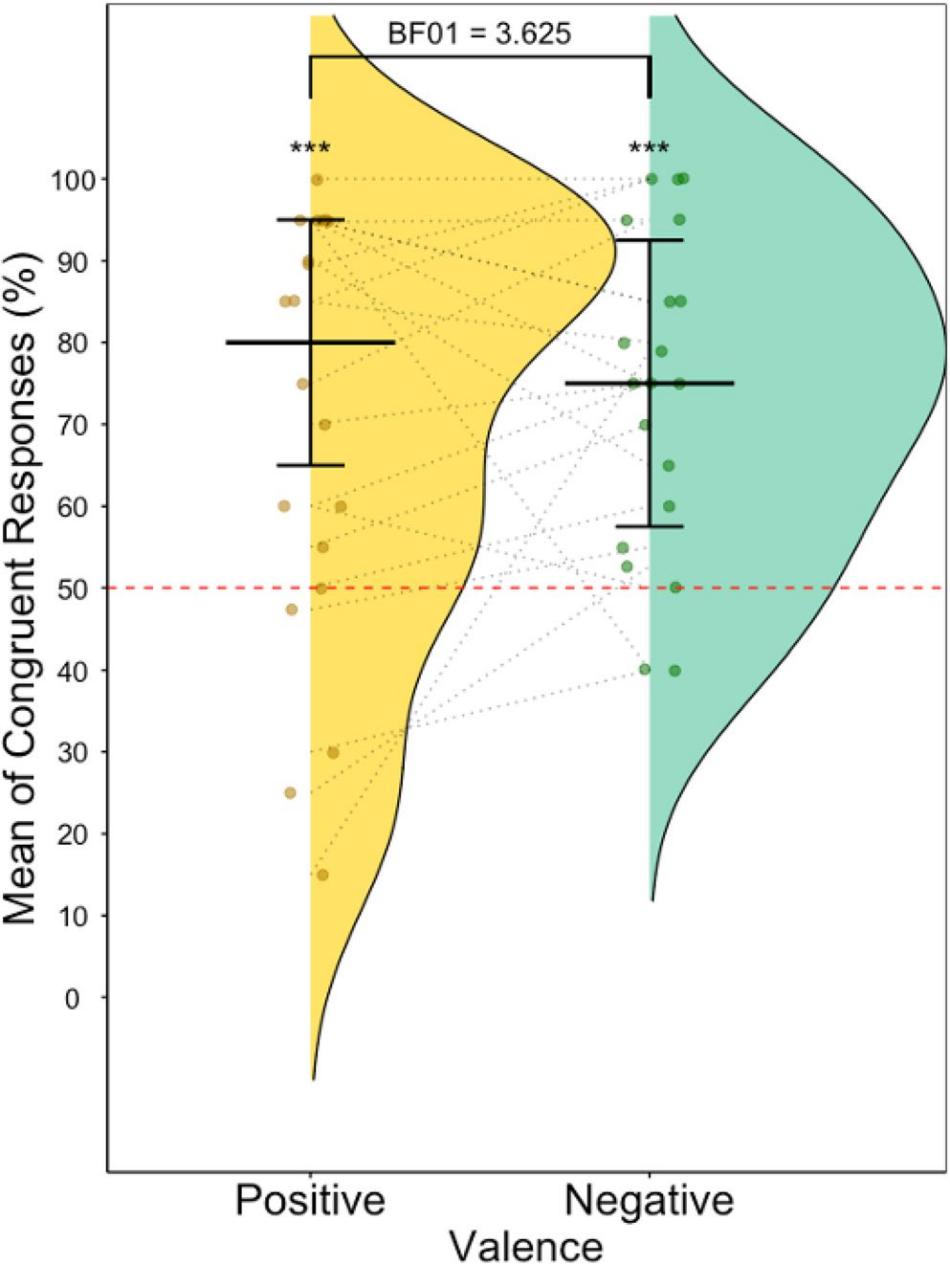
Raincloud plots showing percentage of congruent responses for positive and negative conditions. Red line indicates chance level (50%)

When considering generalization of negative and positive evaluations, one sample t-tests showed that congruent negative emotional reactions were expected (*M* = 73.82%, *SD* = 19.48%) significantly above chance (*t(*19*) =* 5.47, *p <* 0.001, *d =* 1.22, 95% CI [64.71, 82.95]) and that congruent positive emotional responses were also expected (*M* = 70.57%, *SD* = 26.40%) significantly above chance (*t(*19*) =* 3.486, *p <* 0.01, *d =* 0.78, 95% CI [58.22, 82.94]). A paired t-test examining the difference in expectation of congruent responses between positive and negative valence was not significant (*t(*19*) =* 0.62, *p* = 0.544, *d =* 0.14, 95% CI [−7.74, 14.25]) (Fig. 8) and Bayesian tests showed substantial evidence for an absence of difference (BF_01_ = 3.625).

### Discussion experiment 3

As predicted, we found that participants relied on emotional displays when selecting the expected evaluation of novel individuals to unfamiliar actions, displaying an influence of observed emotional displays on their expectations for both positive and negative reactions. Moreover, both positive and negative reactions were generalized to novel individuals. The fact that participants expected either positive or negative evaluations for the very same actions, depending on which valence was observed, provides further evidence against the hypothesis that lack of familiarity with an action automatically triggers an expectation of negative evaluations. This suggests that participants formed expectations based exclusively on the available emotional information. This pattern provides evidence for our interpretation that the absence of generalization for positive reactions to unfamiliar actions in Experiment 2 was not due to a general inability to consistently generalize positive reactions, but rather to the contextual confound created by the simultaneous presence of familiar and unfamiliar actions. In that design, positive reactions to familiar actions did not have a marked conflict with prior-knowledge (i.e., signalling appreciation for the successful end-state of a know action), while positive reactions to unfamiliar actions remained ambiguous and were therefore not generalized. Experiment 3 shows that, once such confound was removed, positive reactions were generalized to the same extent as negative reactions, like confirmed by the Bayesian tests indicating no significant difference in generalization between positive and negative valences.

Thus, even though positive emotional displays may be less crucial for navigating the environment because they do not inform about potential punishment, they are also an important source of information in unfamiliar contexts. Nevertheless, this suggests that the use of negative emotional reactions to create generalized expectations about emotional evaluations of social content seems to be less influenced by contextual cues compared to positive ones.

## General discussion

In line with our key hypothesis, we found that observed emotional expressions displayed in third-party interactions (i.e., affective observation) influence expectations about novel individuals’ evaluations of instrumental actions. Importantly, we found that negative emotional reactions are robustly generalized, being consistently used to form expectations about others’ evaluations when the actions are unfamiliar, but not when they conflict with pre-existing evaluations (familiar actions). This highlights the important role of observed negative reactions in acquiring unfamiliar cultural content. At the same time, the generalization of observed positive reactions to unfamiliar actions seems to be more sensitive to contextual cues. In fact, generalization emerged from positive reactions to unfamiliar actions only in fully unfamiliar contexts - this underscores that both valences can serve as powerful cues for acquiring unfamiliar cultural content, albeit through partially distinct pathways.

Specifically, Experiment 1 showed that although negative emotional displays influence expectations both for familiar and unfamiliar actions, lack of familiarity affects the tendency to generalize negative emotional reactions to instrumental actions; and the neutral baseline excluded the possibility that unfamiliar actions automatically trigger expectations of negative reactions in observers. These findings align with previous research highlighting the crucial role of negative emotional displays in navigating the cultural environment, especially when in ambiguous situations (Bruder et al., 2014). Moreover, negative emotional displays inform observers about potential harmful consequences, such as punishment, which may be particularly crucial when dealing with the unfamiliar (i.e., opaque) cultural content, allowing individuals to detect norm violations and to predict which behaviours to adopt and which to avoid (Lindström & Olsson, 2015).

Experiment 2 replicated the findings of Experiment 1 even in the presence of positive displays. While positive displays influenced expectations for both familiar and unfamiliar actions, they were generalized (i.e., expected above chance) only for familiar actions. We suggest that this asymmetry reflects the ambiguity of interpreting positive reactions in complex contexts: when familiar actions are present, positive responses can be readily understood as simple appreciation for a successful outcome, aligning with prior knowledge (positive feedback to a succesful outcome of a known action) without necessarily conveying culturally shared evaluations. This interpretation highlights a broader difference in how positive and negative displays are used to form expectations. Whereas all emotional displays provide relevant information about the environment (Dukes & Clément, 2019), negative expressions may be particularly salient and less influenced by contextual factors because of their role in avoiding risk (Seymour et al., 2007).

Finally, Experiment 3 resolved this ambiguity by focusing exclusively on unfamiliar actions. In this context, both positive and negative displays were generalized, while again confirming the consistent generalization of negative reactions across all experiments. These findings suggest that, in fully unfamiliar contexts, positive displays are also an important source of information, as they signal potential rewards, thus not being inherently weaker cues, but simply more sensitive to contextual factors (such as how the simultaneous presence of familiar and unfamiliar actions creates uncertainty).

### Future directions

Our findings demonstrate how affective observation is important for cultural learning by allowing naive observers to generalize information to novel individuals within a social group. This capacity plays an important role in the transmission of unfamiliar cultural knowledge. Previous research has focused on the generalizability of unfamiliar cultural content exclusively in relation to intentional communication and teaching (Csibra & Gergely, 2009). Although direct communication is essential in signalling the generalizability of social information (i.e., shared cultural knowledge) (Egyed et al., 2013; Gergely et al., 2007) and the formation of expectations regarding others’ shared evaluations, our findings align with recent suggestions that more implicit mechanisms also facilitate the acquisition and transmission of “opaque” cultural information, even in absence of direct communication (e.g., Affective Social Learning framework) (Clément et al., 2022). Affective observation may represent a foundational layer of cultural learning that relies simply on the association between the valence of an observed emotional display and its target, without necessitating the recognition or retrieval of complex mental states or communicative intentions (Ganzetti et al., 2024). The ability to generalize the rich information provided in emotional displays and to use it to form expectations about the environment, even when they are directed at other individuals, would have been evolutionarily advantageous, as it would allow individuals to access important cultural content without the costs or risks associated with direct interaction. Thus, future research should investigate the relevance of emotional displays in the presence of communicative signals with respect to the generalization of cultural information.

Additionally, our findings raise the possibility that cultural learning from positive emotional expressions depends not only on the expressions themselves but also on observers’ interpretive stance and the contextual balance between familiarity and novelty. In everyday life, novel environments vary considerably in how much familiar scaffolding they provide. For example, when moving to a new workplace or cultural setting, some actions may be entirely unfamiliar, while others resemble known practices but with subtle variations. In such contexts, positive reactions to familiar elements may be interpreted as simple reinforcement of expected outcomes, whereas positive reactions to unfamiliar elements may carry more ambiguous meaning and thus require a stronger “learning stance” from observers. Conversely, environments that are overwhelmingly novel, such as entering a new cultural context with few recognizable reference points, may encourage observers to treat positive reactions as informative signals of culturally shared evaluations. Future research should examine how the density of familiar versus unfamiliar cues shapes the extent to which positive emotional expressions support cultural learning.

Furthermore, our findings are limited to one specific aspect of instrumental actions which can create a culturally opaque context: the lack of familiarity with the end-state of the instrumental action. However, there are many different aspects that may render an action culturally opaque – many cultural practices include unfamiliar means to achieve a certain action or the manipulation of unfamiliar objects with unclear functions. Future research should explore the effect of affective observation on generalization for other elements of action performances that are opaque to a naïve observer. This is particularly interesting for actions with unfamiliar means that are rationally inefficient, which lead to over-imitation of instrumentally irrelevant elements of an action sequence (Gergely et al., 2002; Keupp et al., 2015). It may also be worthwhile to consider that emotional signals may shape the evaluation of causally irrelevant action sequences, affecting whether such sequences are reproduced or avoided (Keupp et al., 2015) in light of a socio-emotional strategy of affiliation (Gruber et al., 2022; Over & Carpenter, 2013).

Importantly, our findings are limited to a very basic kind of emotional expressions consisting of static displays of either positive or negative emotional reactions, future studies should furher investigate generalization of more complex displays of emotion, including less basic and more nuanced emotional reactions (e.g., contempt; jealousy), possibly including the use of dynamic stimuli (i.e., videos).

Moreover, the present study investigated generalization of emotional evaluations across individuals (culturally shared values), but future research should also investigate whether generalization processes may also regard specific sub-groups of individuals; for instance, an emotional evaluation could be generalized only to individuals sharing similar knowledge (e.g., expertise in a certain field) or similar traits. In addition, generalization processes may also take place regarding the actions observed. For instance, whether the negative evaluation of an action extends to actions sharing similar properties (e.g., involving the same objects or belonging to the same “domain”).

Finally, another important factor to consider regarding the effect of emotional displays on generalization is the perceived reliability of the individual displaying the emotion. The perceived reliability of expressers, based either on facial features (Olivola et al., 2014; Oosterhof & Todorov, 2008; Todorov et al., 2008, 2009), or explicit evaluations (e.g., trustworthiness, competence) (Clément, 2010; Harris et al., 2018; Sperber et al., 2010), may make the emotional information even more salient and relevant if an expresser is perceived as being reliable. However, if the expresser is perceived as being unreliable, selfish, incompetent, or untrustworthy, the emotional information might be perceived as being less informative because the source of information would be considered as possibly conveying dubious information.

## Conclusion

Our study is a first step in shedding light on the crucial role played by the observation of emotional displays when navigating unfamiliar cultural environments. Specifically, affective observation may represent a foundational layer of cultural learning that allows individuals to acquire and generalize unfamiliar cultural knowledge to form expectations about the social environment, even in the absence of direct communication. This learning relies on simple and automatic processes and does not involve complex mechanisms typical of instruction-based learning contexts. Observing emotional displays elicited by other individuals’ actions to learn about cultural practices shared within a social group may have played a crucial role in our evolutionary history, as it allows observers to exploit the knowledge of others without the risks or costs associated with direct social interactions. Therefore, humans may have evolved a heightened sensitivity to emotional cues and may possess some predispositions to use such information when dealing with uncertain and ambiguous cultural situations.

## Supplementary Information

Below is the link to the electronic supplementary material.

Supplementary Material 1

## Data Availability

Data and analyses are available on the following link: [https://osf.io/uh4y3/?view\_only=db53af7df0e042b49385050147388440](https:/osf.io/uh4y3/?view_only=db53af7df0e042b49385050147388440).
