# Supplementary material for: Affective observation guides expectations about others’ emotional reactions to unfamiliar action outcomes
Source: Psychol Res. 2025 Nov 11;89(6):171. doi: 10.1007/s00426-025-02206-0 (PMC12605569; doi:10.1007/s00426-025-02206-0)
Supplement: Supplementary file 1 — Supplementary Material 1 [file 426_2025_2206_MOESM1_ESM.docx]

**SUPPLEMENTARY MATERIAL**

This supplementary material includes (1) the report of a pilot study conducted before running the experiments included in the manuscript, (2) exploratory analyses, (3) extra-analysis on perceived efficiency, and (4) links to videos of experimental trials.

1. **Pilot study**

In a pilot study, we tested whether people generalize observed negative emotional expressions targeting instrumental action performances to form expectations about others’ negative evaluations of those actions, i.e., after observing a negative reaction to an action, people expect new individuals to also react negatively when the same action is performed by another individual (congruency of expectation). Moreover, we investigated whether people are more likely to generalize observed reactions when the emotional display targets an unfamiliar compared to familiar action performance.

We predicted participants to generalize observed emotional display (i.e., expect a novel individual to display an emotional expression congruent with the previously observed one) above chance level when it targeted an unfamiliar, but not a familiar action. Furthermore, we predicted that participants would generalize significantly more (measured as a percentage of congruent choices) in the unfamiliar condition compared to the familiar condition. This is because, for unfamiliar actions, the observed emotional reaction would not conflict with prior knowledge about the action performance, thus making it more likely to be used to form an evaluation of the action that could be extended to other individuals.

## **Method**

### **Participants**

We recruited 24 participants (Females = 12, *M_age_* = 25.54 years, *SD* = 6.48) using the Prolific recruitment platform (<https://www.prolific.com>). The criteria to participate included English language proficiency and no diagnosed attentional disorders. One participant was excluded from the analysis due to pre-defined exclusion criteria (i.e., time-limit for submission less than 1h). Participants received monetary compensation (10£/h) through Prolific. A post-hoc power analysis using G*power 3.1, showed that our study was appropriately powered to detect differences in generalization between familiar and unfamiliar actions (1-beta = 0.85; *d* = 0.65; fixed alpha = 0.05).

### **Materials and procedure**

The materials and procedure were the same as Experiment 1 in the manuscript, except the following:

- The observation expresser exhibited only negative reactions instead of neutral and negative.
- When rating the familiarity of actions participants were asked “How familiar is this action?”
- The emotional contagion questionnaire was not present.

### **Design and data analysis**

This experiment employed a within-subjects design, with type of action (familiar or unfamiliar) as a within subject variable and the congruency between the generalization phase expresser’s predicted reaction and the observation phase expresser’s reaction as the dependent variable (i.e., whether the generalization phase expresser’s display was predicted to be negative like the observation phase expresser’s display, or neutral). For each participant, we calculated the percentage of predicted congruent reactions (i.e., percentage of congruent expectation) in each condition (40 trials for the familiar condition; 40 trials for the unfamiliar condition).

To determine whether participants generalized a negative reaction from the first expresser to the second expresser, we conducted two one-sample t-tests to check whether the percentage of congruent expectation in both the familiar and unfamiliar condition was above chance level (i.e., 50%). Additionally, to test whether there were any differences between the familiar and unfamiliar actions, we performed a paired sample t-test comparing percentage of congruent expectation when observing familiar actions versus unfamiliar actions.

When appropriate, we also performed Bayesian tests to evaluate the evidence for the null hypothesis relative to the alternative hypothesis. For all the Bayesian tests performed, the prior was set at 0.707.

Furthermore, we used participants’ ratings of perceived familiarity with the actions to create two subjective categories: actions perceived as being familiar (rating ≥ 4), and actions perceived as being unfamiliar (rating < 4). We conducted the same tests using the subjective categorization of familiar and unfamiliar actions based on participants’ rating.

We excluded 51 trials (out of a total of 1840) with a reaction time less than 200ms or greater than 3000ms).

**Results**

**Manipulation check**

In order to ensure that our action categories accurately reflected participants’ subjective perception of familiarity, we conducted a chi-squared test. The results demonstrated a significant relationship between participants’ categorizations (i.e., whether actions were rated as familiar or unfamiliar) and our experimental manipulation (*X^2^(1)* = 313.05, N = 1840, *p* < 0.001, *V* = 0.41). Specifically, for our set of actions intended to be unfamiliar, participants rated them as being unfamiliar 57.3% of the time, while 32.7% of the time, they judged them to be familiar. Conversely, for actions intended to be familiar, participants rated them as familiar 82.72% of the time, and as unfamiliar 17.28% of the time.

**Generalization based on experimental setting**

One sample t-tests revealed that the percentage of congruent predicted reactions was significantly above chance for unfamiliar actions (*M* = 74.94%, *SD* = 15.84%) (*t(22) =* 7.55*, p <* 0.001*, d =* 1.58), but not for familiar actions (*M* = 57.68%, *SD* = 29.17%), (*t(22) =* 1.26*, p =* 0.22, *d =* 0.26) suggesting that participants generalized negative reactions for unfamiliar but not familiar actions. Additional Bayesian analysis provided anecdotal evidence that participants’ percentage of congruent expectations for familiar actions was mostly at chance level (BF_01_ = 2.263). Moreover, the percentage of congruent expectation was significantly higher in the unfamiliar condition than in the familiar one (*t(22) =* 3.11*, p <* 0.01*, d =* 0.65; S1 Fig.) suggesting that participants generalized negative reactions more for unfamiliar compared to familiar actions.

**
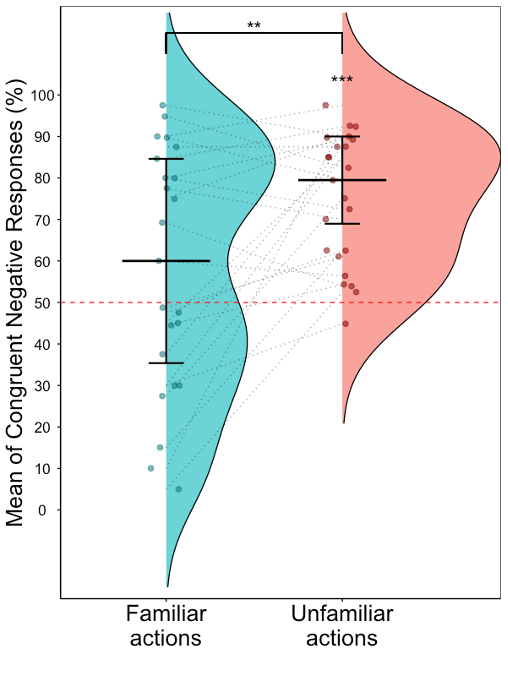
**

**S1 Fig.** **Raincloud plots showing percentage of congruent responses for familiar and unfamiliar conditions.** Red line indicates chance level (50%).

**Generalization based on subjective categorizations of actions’ familiarity**

To confirm that our manipulation was effective, we again performed analyses using the subjective categorizations of actions based on participants’ ratings collected after the experiment. Results showed that percentage of congruent expectations for actions rated as being unfamiliar (*M* = 70.28%, *SD* = 23.21%) was significantly above chance (*t(22) =* 4.19*, p <* 0.001*, d =* 0.87), but not for actions rated as being familiar (*M* = 61.20%, *SD* = 27.72%), (*t(22) =* 1.94*, p =* 0.07, *d =* 0.40). However, expectation of negative responses did not differ significantly between actions rated as familiar and unfamiliar (*t(22) =* 1.21*, p =* 0.39*, d =* 0.25). The Bayesian test indicated anecdotal evidence (BF_01_ = 2.403) that there was no difference in generalization between the two categories.

**Discussion Pilot and rationale of changes in the manuscript studies**

We found that participants generalized negative emotional displays to predict novel observers’ reactions when the target was an unfamiliar but not a familiar action. Lack of familiarity with the target of the reaction led people to rely more on observed emotional displays, whereas familiarity with the target of the reaction conflicted with prior evaluations of the action (i.e., usually not triggering negative evaluations), thus leading to less generalization. These results indicate a strong effect of lack of familiarity on the tendency to generalize negative evaluations of an action performance.

Moreover, even though expectation of negative responses after seeing a negative reaction for familiar actions was not different from chance level, participants displayed different tendencies at the individual level, as clearly illustrated by the distribution in S1 Fig. One subgroup tended to rely on the emotional displays in the familiar condition, whereas another group of people discarded the emotional cue and relying on prior-knowledge. Additionally, some individuals performed at chance level, neither systematically relying on nor completely discarding the emotional cue.

One explanation for over-reliance on negative responses in the familiar condition is that all familiar actions received negative reactions, constantly violating the expectation of a neutral response. This could have led some participants to interpret the emotional display as targeting other elements of the action performances. For example, some participants might have justified the presence of a negative reaction by inferring issues with the means of the action (i.e., how it was performed) rather than the action itself.

For this reason, in the studies included in the manuscript, we decided to Introduce reactions that do not violate prior-evaluations of familiar actions in the observation phase (i.e., neutral responses), as they could increase the extent to which prior knowledge leads to resistance to negative emotional evaluations that target actions that usually do not trigger negative evaluations. Furthermore, since there may be individual differences in the influence of emotional displays on participants’ evaluations, we introduced the emotional contagion questionnaire.

Finally, although we found that subjective categorizations of action familiarity indicated a tendency to generalize reactions about actions perceived as unfamiliar but not familiar (with respect to chance level), we did not find a significant difference between these two categories. This discrepancy may be because a substantial portion (32%) of the actions intended to be unfamiliar were rated as highly familiar. The phrasing of the question used to assess perceived familiarity (i.e., “how familiar is this action?”) might have been ambiguous, leading to a misinterpretation. Some participants might have given higher familiarity scores because they had previously saw these actions in the task or because the objects used were familiar, despite the end-state being “opaque”. Therefore, in the studies reported in the manuscript, when asking participants to rate the familiarity of the performances, we explicitly focussed on familiarity regarding the end-state and goal of the action.

Finally, we included the baseline to have a reference point to measure the effect of observed emotional expressions on expectations (i.e., influence on expectation).

**Experiment 1**

For experiment 1, we (1) performed the same tests of our main analysis using participants’ subjective categorizations of actions, in order to verify that the same pattern of results was also found when using participants’ perceived familiarity, thus strengthening our findings; (2) we performed exploratory analyses to check for a possible correlation between perceived intensity of emotional displays and expectations of congruent reactions. Finally, (3) we performed some exploratory tests to check whether there was a correlation between participants’ emotional contagion scores and their tendency generalize others’ evaluations, especially in relation to familiar actions. In fact, the introduction of this scale was motivated by the goal of investigating potential individual differences in over-generalization (i.e., systematically relying on the emotional cue also when it contrasts with pre-existing evaluations of the target). Nonetheless, we reported the analyses for unfamiliar actions too.

**Generalization based on subjective categorizations of familiarity**

When considering the subjective categorizations of actions based on participants’ ratings, results showed the same pattern found using the experimental setting categories: to examine the interaction between valence and type of action with respect to generalization our 2 x 2 ANOVA revealed a significant main effect of valence (F(1, 23) = 6.67, *p* < 0.05, *ηp2* = 0.22), a no effect of action subjective categorization (F(1, 23) = 0.44, *p* = 0.51, *ηp2* = 0.02) and a significant interaction between subjective action categorization and valence (F(1, 23) = 25.13, *p* < 0.001*, ηp2* = 0.52) on percentage of congruent expectations.

Participants’ percentage of congruent expectations for negative emotional displays targeting actions rated as being unfamiliar (M = 73.92%, *SD* = 22.75%) was significantly above chance (*t*(23) = 5.15, *p* < 0.001, *d* = 1.05), while it was not the case for actions judged to be familiar (M = 44.75%, *SD* = 33.05%), (*t*(23)) = -0.78, *p* = 0.44). The Bayesian test indicated moderate evidence (*BF_01_* = 3.542) that congruent expectations of negative emotional displays for actions perceived as familiar was not different from the level of chance. The difference in participants’ generalization of negative responses between the two categories of perceived familiarity was also significant (t(23) = 4.89, *p* < 0.001, *d* = 0.99). Furthermore, expectation of congruent neutral responses was significantly above the level of chance for actions perceived as familiar (M = 84.26%, *SD* = 13.56%) (*t*(23) = 12.373, *p* < 0.001, *d* = 2.53), but not for those perceived as unfamiliar (M = 57.64%, *SD* = 26.93%) (t(23) = 1.389, *p* = 0.178, *d* = 0.28); the Bayesian test provided anecdotal evidence for generalization of neutral responses targeting perceived unfamiliar actions at the level of chance (*BF_01_* = 1.992). Results also showed a significant difference in generalization of neutral responses between perceived familiar and perceived unfamiliar actions (t(23) = 4.56, *p* < 0.001, *d* = 0.93). Moreover, results indicated a significant difference in generalization of congruent negative and neutral reactions for actions perceived as unfamiliar (t(23) = 2.51, *p* < 0.05, *d* = 0.51) and for actions perceived as being familiar (t(23) = -5.09, *p* < 0.001, *d* = 1.04).

**Generalization and emotional intensity ratings**

We performed a Pearson correlation test between intensity ratings (continuous variable ranging from 1 to 7) and expectation of congruent negative emotional displays (i.e., congruent or incongruent choice). The tests showed that there was not a significant correlation between perceived intensity of negative emotional displays and the tendency to systematically expect a congruent reaction (*r* = 0.02, *p* = 0.47); also when considering unfamiliar (*r* = 0.001, *p* = 0.97) and familiar actions (r = 0.06, *p* = 0.21) separately. We then included all displays’ ratings (neutral and negative) in the analysis, considering familiar and unfamiliar conditions separately, in order to check whether, in line with our main results, intensity of reactions was positively correlated with expectation of congruent reactions in the unfamiliar condition and negatively correlated with it in the familiar condition. The results of the tests showed a significant positive correlation between perceived intensity of the emotional displays for the unfamiliar condition (*r* = 0.14, *p* < 0.001) and a negative correlation for the familiar condition (*r* = -0.34, *p* < 0.001), in line with our main predictions (i.e., negative emotional displays are more intense than neutral ones). These results further strengthen the claim that our manipulation correctly worked.

**Generalization and emotional contagion scores**

We performed some tests to investigate possible correlations between participants’ emotional contagion scores and percentage of congruent responses. The score was obtained by summing the scores, ranging from 1 to 5, for each question (*n* = 15) included in the questionnaire (min. score = 15 – max. score = 75). Since the study was not designed to test correlations, we were likely underpowered, so we decided not to report the analyses in the paper.

The correlation test did not reveal a significant relationship between participants’ emotional contagion scores and congruent expectations in the familiar condition (*r* = 0.27, *p* = 0.21). Regarding unfamiliar actions, we found a significant positive correlation between emotional contagion scores and congruent expectations (*r* = 0.41; *p* = 0.044). In addition to the reasons reported at the beginning of this paragraph, we also did not report these results in the paper as (a) it is not very informative, and (b) the significance of the correlation may be due to one outlier that we noticed when plotting the data

**Experiment 2**

Same exploratory analyses conducted for experiment 1, with positive instead of neutral displays.

**Generalization based on subjective categorizations of familiarity**

When considering the subjective categorizations of actions based on participants’ ratings, results showed the same pattern found using the experimental setting categories: to examine the interaction between valence and type of action with respect to expectation of congruent reactions our 2 x 2 ANOVA revealed a no main effect of valence (F(1, 19) = 0.026, *p* = 0.87, *ηp2* = 0.0001), a no effect of action subjective categorization (F(1, 19) = 0.20, *p* = 0.66, *ηp2* = 0.01) and a significant interaction between subjective action categorization and valence (F(1, 19) = 9.78*, p* < 0.01, *ηp2* = 0.12) on percentage of congruent expectations.

Participants’ percentage of congruent expectations for negative emotional displays targeting actions rated as being unfamiliar (M = 79.52%, *SD* = 22.01%) was significantly above chance (t(19) = 5.99, *p* < 0.001, *d* = 1.34), while it was not the case for actions judged to be familiar (M = 48.10%, SD = 36.64%), (t(19)) = -0.23, *p* = 0.82, *d* = 0.05). The Bayesian test indicated moderate evidence (*BF_01_* = 4.201) that expectation of congruent reactions after observing negative emotional displays for actions perceived as familiar was not different from the level of chance. The difference in participants’ expectations of congruent negative responses between the two categories of perceived familiarity was also significant (t(19) = 4.23, *p* < 0.001, *d* = 0.95). Furthermore, expectation of congruent positive responses was significantly above the level of chance for actions perceived as familiar (M = 85.01%, SD = 14.24%) (t(19) = 10.99, *p* < 0.001, *d* = 2.46), but not for those perceived as unfamiliar (M = 57.31%, *SD* = 29.03%) (t(19) = 1.13, *p* = 0.274, *d* = 0.25); the Bayesian test provided anecdotal evidence for expectation of congruent positive responses targeting perceived unfamiliar actions at the level of chance (*BF_01_* = 2.466). Results also showed a significant difference in expectation of congruent positive responses between perceived familiar and perceived unfamiliar actions (t(19) = 4.56, *p* < 0.001, *d* = 0.93). Moreover, results indicated a significant difference in expectation of congruent negative and positive reactions for actions perceived as unfamiliar (t(19) = 2.98, *p* < 0.001, *d* = 0.67) and for actions perceived as being familiar (t(19) = -4.72, *p* < 0.001, *d* = 1.05).

**Generalization and emotional intensity ratings**

Although we did not have precise predictions, in our exploratory analysis we performed a Pearson correlation test between intensity ratings (continuous variable ranging from 1 to 7) and congruent or incongruent expectations. The tests showed that there was a significant negative correlation between perceived intensity of negative emotional displays and the tendency to expect a congruent expectation (*r* = -0.14, *p* < 0.001). We then analysed separately percentage of congruent expectations of negative displays considering familiar and unfamiliar actions. The results of the tests showed a significant negative correlation between perceived intensity of the emotional displays for the unfamiliar condition (*r* = -0.12, *p* < 0.05) and a negative correlation for the familiar condition (*r* = -0.16, *p* < 0.01).

Finally, we analysed expectation of congruent positive displays finding no significant correlations with perceived intensity of the emotional displays (*r* = 0.003, *p* = 0.94). We then considered familiar and unfamiliar actions separately. The results of the tests showed the absence of a significant correlation between perceived intensity of positive emotional displays and expectation of congruent reactions, both for unfamiliar (*r* = 0.025, *p* = 0.62) and familiar actions (*r* = 0.046, *p* = 0.37).

**Generalization and emotional contagion scores**

We performed some tests to investigate possible correlations between participants’ emotional contagion scores and percentage of congruent responses. Emotional contagion scores were created like in Experiment 1.

The correlation test did not reveal a significant relationship between participants’ emotional contagion scores and expectations of congruent reactions for negative emotional displays in the familiar condition (*r* = 0.26, *p* = 0.26) and in the unfamiliar condition (*r* = 0.34, *p* = 0.14). Regarding positive displays, we did not find a significant positive correlation between emotional contagion scores and expectation of congruent reactions for unfamiliar actions (*r* = 0.21, *p* = 0.38).

**Experiment 3**

Since this experiment included only unfamiliar actions, we performed exploratory analyses only regarding possible correlations between (a) participants’ perceived intensity of the emotions displayed and expectation of congruent reactions, and (b) emotional contagion scores and expectation of congruent reactions.

In fact, although the main reason why we introduced the emotional contagion scale was to investigate potential individual differences in over-relying on emotional cues targeting familiar actions, we also checked unfamiliar actions in all experiments. Thus, we decided to report it here also.

**Generalization and emotional intensity ratings**

We performed a Pearson correlation test between intensity ratings (continuous variable ranging from 1 to 7) and expectation of congruent negative emotional displays (i.e., congruent or congruent choice). The tests showed that there was not a significant correlation between perceived intensity of emotional displays and the tendency to expect congruent reactions (*r* = 0.02, *p* = 0.47); also when considering negative (*r* = -0.01, *p* = 0.80) and positive emotional displays (*r* = 0.04, *p* = 0.29) separately.

**Generalization and emotional contagion scores**

We investigated possible correlations between participants’ emotional contagion scores and percentage of congruent responses. Emotional contagion scores were created like in Experiment 1.

The correlation test did not reveal a significant relationship between participants’ emotional contagion scores and expectation of congruent responses when considering positive and negative displays together (*r* = 0.12, *p* = 0.61).

We also did not find any significant correlations when considering positive (*r* = 0.38, *p* = 0.10) and negative (*r* = -0.28, *p* = 0.24) displays separately.

**Perceived efficiency check**

We have conducted an additional check to ensure that perceived efficiency of unfamiliar action outcomes did not influence the tendency to generalize observed emotional displays. We collected perceived efficiency scores regarding our set of unfamiliar actions, and we have used these scores to check for an effect on our main findings.

## **Method**

### **Participants**

We recruited 50 participants (Females = 25, *M_age_* = 25.48 years, *SD* = 7.53) using the Prolific recruitment platform (<https://www.prolific.com>). The criteria to participate included English language proficiency and no diagnosed attentional disorders. Participants received monetary compensation (12£/h) through Prolific.

### **Materials and procedure**

Participants were presented with the set of unfamiliar actions and were asked to rate the perceived efficiency of the observed outcome using a slider ranging from 1 (“very inefficient”) to 7 (“very efficient”).

We have used these scores to test whether perceived efficiency of the action outcome influenced participants’ tendency to expect congruent emotional reactions in our study.

Specifically, for each condition of our set of Experiments, we have conducted a generalized linear mixed model with expectation of congruent response as a dependent variable and perceived efficiency score of the action observed as a fixed effect. A logit link function was applied. Because we have done the comparison for each level of the design for each experiment, we applied Bonferroni correction (for instance, Experiment 1 includes a separate model for negative and neutral responses, so we corrected for two comparisons).

**Negative reactions (Exp.1-2-3)**

In Experiment 1, we found no significant effect of perceived efficiency on participants’ tendency to generalize negative responses (*β =* -0.34, *z* = -1.563**,** *p* = 0.24). As shown in S2 Fig.1A, the observed slope is driven by an outlier.

A similar pattern was observed in Experiment 2, where perceived efficiency again did not have a significant effect on expectation of congruent negative reactions (*β =* -0.43, *z* = -1.813**,** *p* = 0.14), as illustrated in S2 Fig.2A.

Experiment 3 also showed no significant effect of perceived efficiency on expectation of congruent negative responses (*β =* -0.40, *z* = -1.814**,** *p* = 0.14). The scatter plot for Experiment 3 (S2 Fig.3A) showed some differences compared to Experiments 1 and 2, likely due to the exclusive presentation of unfamiliar actions, which prompted participants to adopt varying strategies.

**Neutral baseline (Exp.1)**

In the neutral baseline condition, as expected, the model displayed a significant effect of perceived efficiency on expectation of congruent neutral responses (*β =* 0.65, *z* = 3.421**,** *p* < 0.01; S2 Fig.1B). Since neutral displays are less informative than emotional reactions, their expectation was more likely to be influenced by subjective evaluations of the action. Participants expected neutral responses more consistently when the action was perceived as being efficient, and less when it was perceived as being inefficient.

**Positive displays (Exp.2-3)**

In Experiment 2, the model revealed a significant effect of perceived efficiency on expectation of congruent positive responses (*β =* -0.50, *z* = 2.269, *p* = 0.046; S2 Fig.2B).

This supports our interim discussion of Experiment 2 in the manuscript, suggesting that the presence of familiar actions likely made positive displays ambiguous.

Participants may have interpreted these displays as evaluations of the successful completion of an action goal rather than as cultural evaluations.

Experiment 3 revealed no main effect of perceived efficiency on expectation of congruent positive responses (*β =* 0.30, *z* = 1.278, *p* = 0.40; S2 Fig. 3B). This suggests that when exclusively considering unfamiliar actions, the ambiguity of interpreting positive displays disappears, leading to generalization of positive responses regardless of perceived efficiency.

**General observations**

Overall, these results highlight that perceived efficiency did not significantly affect participants’ expectations of congruent negative responses. However, perceived efficiency appeared to play a role in shaping expectations for neutral and positive displays in the context of both familiar and unfamiliar actions. Importantly, when focusing exclusively on unfamiliar actions, even expectations of congruent positive responses were not influenced by perceived efficiency. These findings suggest that perceived efficiency is an important aspect of observers’ prior evaluations, influencing expectations of reactions displayed by novel observers. However, it does not account for our main findings, particularly regarding negative responses, which were our primary condition of interest.


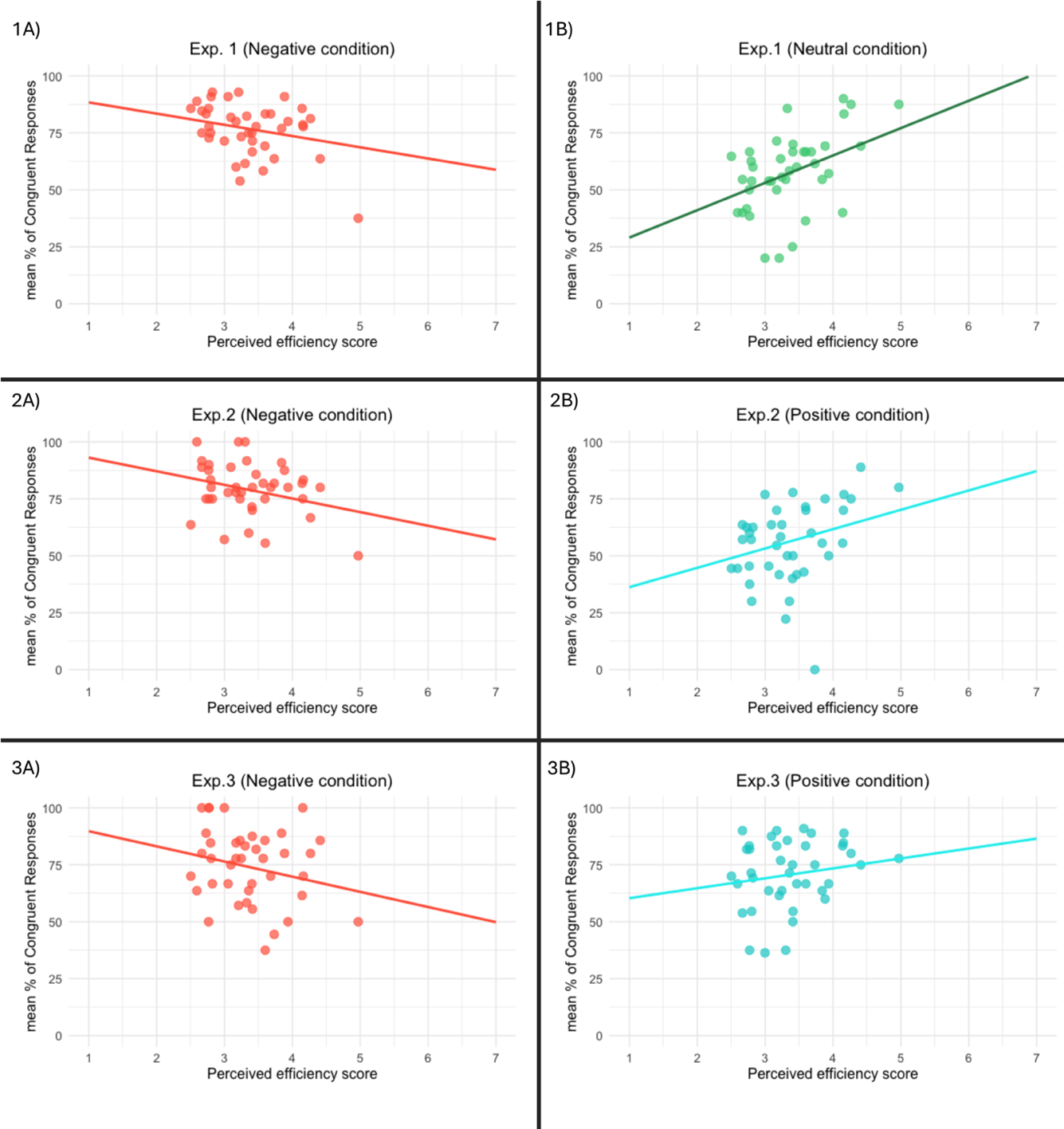


**S2 Fig.** **Relationship between perceived efficiency scores and expectation of congruent responses in each condition.**

**AMPLE VIDEOS OF TRIALS:**

The videos can also be downloaded with all the other study material using this link: [**https://osf.io/uh4y3/?view_only=db53af7df0e042b49385050147388440**](https://osf.io/uh4y3/?view_only=db53af7df0e042b49385050147388440)

In the folder, you can find the following trial-videos:

- **Negative unfamiliar (Exp.1)**
- **Negative familiar (Exp.1)**
- **Negative unfamiliar (Exp.2; 3)**
- **Positive unfamiliar (Exp.2; 3)**
- **Positive familiar (Exp.2)**
